# Does Acidification Affect Urinary Creatinine in Dairy Cattle?

**DOI:** 10.3390/ani14020315

**Published:** 2024-01-19

**Authors:** Tommaso Danese, Maria Chiara Sabetti, Nicolò Mezzasalma, Marica Simoni, Cecilia Quintavalla, Federico Righi

**Affiliations:** Department of Veterinary Sciences, Parma University, 43126 Parma, Italy; tommaso.danese@unipr.it (T.D.); nicolo.mezzasalma@unipr.it (N.M.); marica.simoni@unipr.it (M.S.); cecilia.quintavalla@unipr.it (C.Q.); federico.righi@unipr.it (F.R.)

**Keywords:** urinary nitrogen, urine acidification, urine spot sampling, urinary markers in lactating cows

## Abstract

**Simple Summary:**

Creatinine is a marker commonly employed to quantify the amount of urine produced by dairy cattle, when a representative spot urine sample is collected. Combined with urine nitrogen content, the latter allows the measurement of the urinary nitrogen excretion, which is needed to partially assess the environmental impact of dairy farming. However, when urine is sampled, a quota of ammonia nitrogen tends to volatilize, potentially leading to an underestimation of the content of this element. This issue is normally prevented by sample acidification with sulphuric acid which, along with diluting the sample itself, could chemically alter the creatinine content, leading to bias in N excretion quantification. The purpose of our study was to assess whether acidification could affect the creatinine content and detection in urine, and the results demonstrate that acidification can influence its measurement in urine samples from dairy cattle.

**Abstract:**

Nitrogen content in urine plays a crucial role in assessing the environmental impact of dairy farming. Urine acidifications avoid urine nitrogen volatilization, but potentially lead to a degradation of creatinine, the most dependable marker for quantifying total urine excretion volume, affecting its measurement. This study aimed to assess how acidifying urine samples affects the concentration and detection of creatinine in dairy cattle. In this trial, individual urine samples from 20 Holstein lactating dairy cows were divided into three subsamples, allocated to 1 of 3 groups consisting of 20 samples each. Samples were immediately treated as follows: acidification with H_2_SO_4_ (1 mL of acid in 30 mL of sample) to achieve a pH < 2 (Group 1)); addition of an equal volume of distilled water (1 mL of distilled water in 30 mL of sample) to investigate dilution effects (Group 2); or storage without any acid or water treatment (Group 3). An analysis of creatinine levels was carried out using the Jaffe method. The Friedman test was employed to compare urine groups across treatments, and the Bland–Altman test was used to assess the agreement between measurements in Group 1 and Group 3. Urinary creatinine values were statistically different (*p* < 0.001) between Group 1 (median 48.5 mg/dL; range 36.9–83 mg/dL), Group 2 (median 47.5 mg/dL; range 36.5–80.7 mg/dL), and Group 3 (median 48.9 mg/dL, range 37.2–84). Bland–Altman analysis demonstrates agreement between Group 3 and Group 1. The measurement of urinary creatinine using the Jaffe method is affected by sample acidification, but the use of creatinine as a marker for total urine output could remain a viable tool when urine samples are acidified.

## 1. Introduction

Creatinine is a catabolic residue of creatine metabolism, excreted by the kidney in the urine, particularly suitable to be used as a marker for the estimation of total urine output in dairy cows. This attitude relates to its spontaneous, irreversible, and non-enzymatic conversion from creatine catabolism in skeletal muscles and to excretion at a constant rate [1]. Moreover, creatinine is only marginally influenced by muscle mass and nutrients intake, referring in particular to proteins [2], non-protein nitrogen [3], and non-fibre carbohydrates [4]. Additionally, creatinine concentration depends also on the body hydration status, and, therefore, it has a diurnal excretion reflecting cow-to-cow variability [5,6]. To avoid the effect of these fluctuations on the estimation of urinary output, total urine collection is addressed, but different sampling protocols have also been developed. Total urine collection is considered the gold standard for urine studies. Conversely, urine spot sampling is an indirect measure based on an analytical equation, and therefore less accurate than total collection. However, there are certain conditions (i.e., grazing herds) which force researchers toward the use of these sampling protocols. The literature reports several different approaches to spot urine sampling, varying from a single 6–8 h post-feeding sampling [7] to 12 sampling events in 3 days [5]. Regardless of sampling times, total urine output is calculated as the product of the body weight of the animal considered (BW, kg) and a fixed coefficient (mg/kg BW), divided by the individual urine creatinine content (mg/L). In the formula, the fixed coefficient may vary among authors and studies [5,8]. The use of the latter formula for total urine output quantification is frequent in the dairy industry, specifically to measure urinary Nitrogen (N) excretion. In this application, a critical point is to avoid N losses from the sample, which can occur due to ammonia N (NH_3_) volatilization; for this reason, urine samples are usually acidified immediately after the collection. A widely used method is to add inorganic acids directly to the urinary samples with the objective to drop the pH level under 2 [3]. This also avoids the alteration of the samples deriving from the contamination of urine by faecal bacteria and the related metabolites, which often occurs in the field [7]. It is hypothesized that urine acidification could impact creatinine stability, detection, or measurement due to the potential chemical alteration and physical dilution effect, which a strong acid could apply to creatinine, altering its potential as a marker of total urine output. Thus, the aim of this trial was to define if urine creatinine content measurement is affected by sample acidification.

## 2. Materials and Methods

Individual urine samples from 20 Holstein lactating dairy cows of the teaching dairy barn of Dipartimento di Scienze Medico Veterinarie, Università di Parma, were collected. The cows belonged to the same dietary group and the average group feed intake and milk yield were 26 and 29 kg/d, respectively. Due to the traditional hay-based feeding system used in the teaching dairy barn, dietary ingredients were individually sampled once when fed to the animals. Dry matter (DM) content of the dietary components was measured by drying the sample at 103 °C overnight. Neutral detergent fibre (aNDF) of the dietary components was determined using heat-stable amylase but no sodium sulphite and expressed inclusive of the residual ash [9]. The N content of the dietary components was determined by the combustion digestion of the sample at 900 °C in excess of oxygen by Dumatherm (Gerhardt GmbH & Co, Königswinter, Germany), as described by [10] Crude protein (CP) was calculated from N using the fix coefficient as described in [11]. The cows were fed a hay-based diet, including alfalfa (aNDF 54% of DM, CP 15% of DM), mixed hay (aNDF 52% of DM and CP 13% of DM), and a commercial complementary feedstuff (aNDF 16% of DM and CP 17% of DM). Urine sampling was performed at 10 am, 4 h after feeding, during urination induced by perineal massage: the vulva and the perineal region were wiped with a clean paper towel to remove faecal residues, and the first millilitres of urine were discharged before sampling. Each cow was sampled once and the sample was immediately divided into 3 subsamples: a first subsample was acidified to obtain a pH lower than 2 (Group 1) by means of 50% sulfuric acid (H_2_SO_4_, 1 mL in 30 mL of urine); a second was added with the same volume of distilled water (1 mL in 30 mL of urine) to study the dilution effect (Group 2); and the last one was stored as is without acid or water (Group 3). Urine samples were promptly refrigerated at 4 °C and transferred for urinalysis to the Clinical Pathology Laboratory of the Ospedale Veterinario Universitario Didattico (OVUD) of Università di Parma, located 5 km apart from the herd. The samples were processed, according to quality standard procedures, within two hours from the collection. For processing, after a visual assessment of appearance and colour to verify the absence of contaminations or alteration (i.e., haemolysis) of the samples, 5 mL of urine was transferred into conical tubes. Tubes were centrifugated at 500× *g* at 8 °C for 10 min to avoid inhomogeneity of the sample. Chemical analysis and urine specific gravity (USG) were assessed on supernatant. Creatinine analyses were conducted using an automated chemistry analyser (BT3500 Biotecnica Instruments, Rome, Italy) with a specific reagent through the Jaffe method. The density of the samples was evaluated through USG by means of a hand refractometer (American Optical, Buffalo, NY, USA) to evaluate the dilution effect.

Statistical analyses were performed using Medcalc (MedCalc Statistical Software version 18.10.2; Ostend, Belgium). Normality was assessed graphically and by using the Shapiro–Wilk test. Since creatinine and USG values were not normally distributed, the data were expressed as median and range (minimum–maximum value). The Friedman test was used to compare creatinine and USG between groups for treatments (Group 1 vs. Group 2 vs. Group 3). A Bland–Altman test was applied to calculate the agreement between Group 1 and Group 3 for creatinine and 95% limits of agreement were calculated. Furthermore, a linear regression analysis was performed to describe the relationship between creatinine in Group 1 and creatinine in Group 3. The statistical significance was set at *p ≤* 0.05. 

## 3. Results

**Figure 1:**
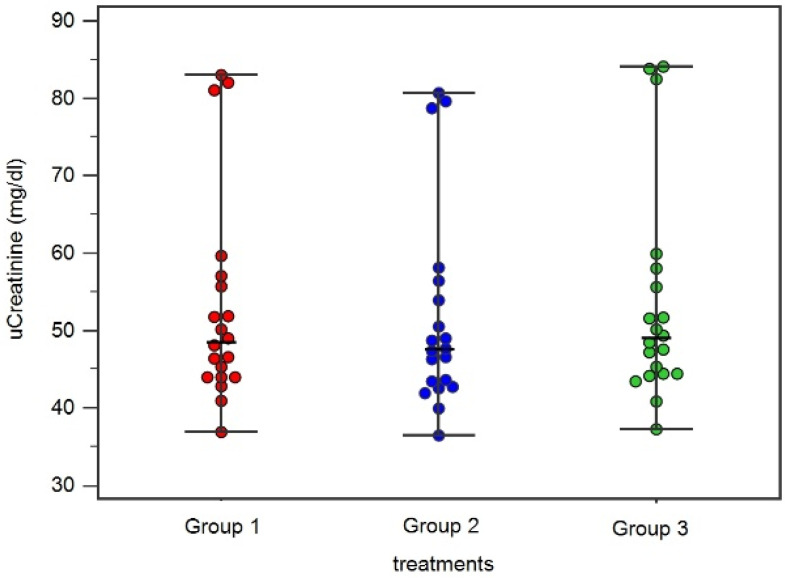
Dot plot showing results of urinary creatinine comparison between acidified urine (Group 1), diluted urine (Group 2), and urine without any acid or water treatment (Group 3). Upright bars represent minimum and maximum values, while horizontal lines (central bars) represent median value.

**Figure 2:**
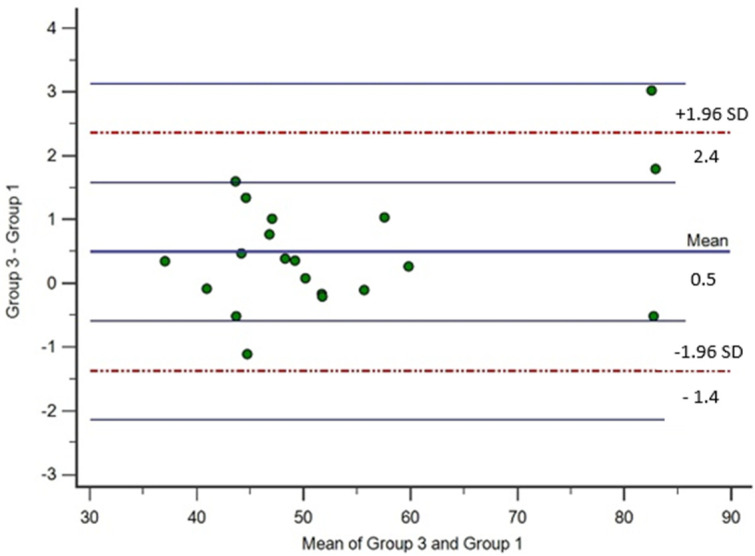
Limits of agreement (Bland–Altman) plot showing differences concerning urinary creatinine measured in acidified urine (Group 1) and in non-acidified urine (Group 3).

Urinary creatinine values were statistically different (*p* < 0.001) between Group 1 (median 48.5 mg/dL; range 36.9–83 mg/dL), Group 2 (median 47.5 mg/dL; range 36.5–80.7 mg/dL), and Group 3 (median 48.9 mg/dL, range 37.2–84; Figure 1). The USG resulted different between groups (*p* < 0.001), with a median of 1026 in Group 1 (range 1022–1031), a median of 1025 for Group 2 (range 1020–1030), and a median of 1025 for Group 3 (range 1022–1031). The Bland–Altman analysis showed agreement between the creatinine measured between Group 3 and Group 1 (Figure 2). The mean difference (95% CI) between creatinine measured in Group 3 and Group 1 was 0.48 (0.04 to 0.93) and the upper and lower limits of agreement were 2.35 and −1.4, respectively. The regression showed a strong linear relationship between creatinine in Group 1 and in Group 3 (R^2^ of 0.99, intercept 1.017, slope of 0.97, and *p <* 0.001). 

## 4. Discussion

Creatinine in blood is formed by the degradation of creatine phosphate, an important energy reserve in muscle tissue metabolism. After its formation, it is filtered and excreted by the kidneys through the urine [11]. Creatinine is widely used as a marker for the measurement of urinary volume through spot sampling protocols [8]. Through the estimation of urine volume, it is possible to assess the excretion of dietary metabolites and components, of which N and purine derivatives are the main examples. Thus, an accurate measurement of creatinine in urine is fundamental for the estimation of other parameters. 

Volatilization of NH_3_ depends on N urea in 60 to 90% of cases, but also to a lesser extent on other important N compounds such as hippuric acids, allantoin, uric acid, xanthine, and creatinine. In order to avoid this, the acidification of samples is used for research purposes in dairy nutrition [12]. 

Furthermore, sample preservation plays a central role in the measurement of creatinine in acidified urine. In fact, acidified urine preserved without freezing, especially at room temperature, can increase the conversion of creatine into creatinine [13]. A recent study demonstrated that storing samples at temperatures that promoted urine freezing (−20 °C and −40 °C) is the best solution when creatinine analysis is not performed within 15 days [14]; for this reason, samples were refrigerated and analysed within two hours of collection. Considering our study outcomes, both acidified samples (Group 1) and diluted samples (Group 2) demonstrated creatinine values lower than the non-acidified ones (Group 3). This observation leads to the hypothesis that a dilution effect was induced by the volumes introduced during the experimental procedures. Consequently, the experimental conditions, notably the acidification and dilution processes, influenced creatinine levels. In contrast, the acidified samples (Group 1) had a higher concentration of creatinine compared to the diluted samples (Group 2). This variation in creatinine levels could be related to the acid’s impact on the conversion of creatine to creatinine, as delineated in prior research [13]. Despite this distinction, even with a difference observed between the acidified (Group 1) and diluted (Group 3) samples, the creatinine levels in the acidified samples (Group 1) remained lower than those in the non-acidified samples (Group 3). It is important to recognize that the implications of our findings, while possessing analytical and statistical significance, necessitate contextualization within a broader biological framework. It is considered that this could marginally impact the calculation of total urine volume output. However, such considerations do not exclude the utilization of creatinine as a reliable marker for total urine output. Further investigations and a better comprehension of these dynamics are needed to enhance the practical applicability of our findings in a field context.

Our results show satisfactory agreement between the results of creatinine analysis performed on acidified and non-acidified samples (Group 1 vs. Group 3). The slight average discrepancy between the methods confirms the statistical difference of our results. 

The regression showed a strong linear relationship between creatinine in acidified samples (Group 1) and non-acidified ones (Group 3). Even if there is a statistical difference between the treatments reported and thus the data are not comparable, in the case of the use of acidified urines, it is likely that the addition of acid will homogenously act on the samples. 

Thus, for the biological, nutritional, and environmental purposes of our work, we still consider creatine in acidified urines as a valuable marker for total urine output quantification when spot sampling is performed and comparison between groups is the main goal of the study. However, the potential bias this work has revealed must be considered. 

Our study has several limitations. Firstly, there is a low number of samples included for each group. Additionally, we did not conduct multiple measurements on the same sample, which would have enabled us to further explore the significance of the differences found. Nevertheless, we believe that our brief communication lays the foundation for further studies and draws attention to the importance of exploring the role of acidification in samples intended for livestock measurements.

What we reported in this communication is to be considered as preliminary data. It will be necessary to include a higher number of observations and apply an intra- and inter-assay trial to further confirm our findings. 

## 5. Conclusions

In conclusion, the practice of acidifying urine to avoid N volatilization can be considered useful for environmental and nutritional evaluation purposes, but further considerations need to be made since the effect of acidification or dilution on creatinine cannot be completely excluded. 

## Data Availability

The data presented in this study are available free of charge for any user on request from the corresponding authors.
